# The complete chloroplast genome sequence of *Actinidia eriantha*

**DOI:** 10.1080/23802359.2019.1623111

**Published:** 2019-07-10

**Authors:** Ping Tang, Ruinan Shen, Ruiwen He, Xiaohong Yao

**Affiliations:** a Key Laboratory of Plant Germplasm Enhancement and Speciality Agriculture, Wuhan Botanical Garden, the Chinese Academy of Sciences, Hubei, China;; b College of Life Sciences, University of Chinese Academy of Sciences, Beijing, China

**Keywords:** *Actinidia eriantha*, chloroplast genome, Actinidiaceae, phylogenetic analysis

## Abstract

The complete chloroplast (cp) genome sequence of *Actinidia eriantha* was sequenced and assembled using Illumina paired-end data. The cp genome from *A. eriantha* is 156,964 bp in length, composed of a pair of 23,892 bp inverted repeat regions (IR) separated by a large single copy region (LSC) of 88,639 bp and a small single copy region (SSC) of 20,541 bp. The cp genome contained 113 unique genes, including 79 protein-coding genes, 30 tRNA genes, and four ribosomal RNA genes. The phylogenetic position of *A. eriantha* based the cp genome data was sister to the group *A. rufa*, *A. deliciosa*, and *A. chinensis*.

*Actinidia* Lindl. belong to family Actinidiaceae and consists of 54 species and 21 varieties (Kumar et al. 2016) found mainly in East and South Asia. *Actinidia eriantha* is endemic to China and has been listed in the second category of key protected wild plants (Guo et al. 2017). The fruit traits of *A. eriantha* have several advanatges, such as high vitamin C, easiness of pelling the pericarp and good storage (Zhong et al. 2012). It is recognized as a valuable species for commercial kiwifruit improvement as well as having been used in traditional Chinese medicine (Zhong et al. 2012; Sun et al. 2015). However, little is known the genetic diversity and genetic differentiation among the populations of *A. eriantha*. Such information is essential for developing optimum conservation and management strategies for *A. eriantha*. In this study, we report the first complete plastome of *A. eriantha*, and assessed its phylogenetic position within Actinidaceae.

One individual of *A. eriantha* was sampled from National Kiwifruit Germplasm Genebank of China, Wuhan Botanical Garden, the Chinese Academy of Sciences (30°33′48.6″ N, 114°25′1.2″ E). Total DNA was isolated using the DNeasy plant Mini Kit (Quiagen, Carlsbad, CA) and stored in a DNA bank in the Wuhan Botanical Garden, the Chinese Academy of Sciences. The strategy for sequencing, assembly and annotation the chloroplast genome was adapted from Yao et al. (2015). The sequence of chloroplast genomes was deposited in GenBank (accession numbers KY100978).

The complete cp genome sequence of *A. eriantha* is 156,964 bp in length. The complete cp genome exhibit the typical quadripartite structure, including a pair of IRs (23,892 bp) separated by the LSC (88,639 bp) and SSC (20,541 bp) regions. The cp genomes encode an identical set of 113 predicted functional genes when duplicated genes are calculated only once, including 79 protein-coding genes, 30 transfer RNA (tRNA) genes, and four rRNA genes. Five protein-coding (i.e. rps7, ndhB, ycf15, ycf2, and rps12), eight tRNA (i.e. trnH-GUG, trnI-CAU, trnLCAA, trnI-GAU, trnV-GAC, trnA-UGC, trnR-ACG, and trnN-GUU) and all four rRNA genes are duplicated in the IR regions, and one tRNA gene [*trnf*M (CAU)] is duplicated in the LSC regions. The LSC region contains 62 protein-coding and 21 tRNA genes, and the SSC region contains 12 protein-coding and one tRNA gene. The GC content of *A. eriantha* cp genomes is 37.2%, which is similar to the other reported *Actinidia* cp genomes (Yao et al. 2015; Lan et al. 2018). The GC content of the IR regions (43.1%) is higher than that of the LSC and SSC regions (35.4% and 31.1%, respectively).

To confirm the phylogenetic position of *A. eriantha*, nine complete chloroplast genome sequences of Actinidiaceae were aligned using MAFFT v.7 (Katoh and Standley 2013) and maximum parsimony (MP) analysis was conducted using with PAUP software (version 4.0b10) with 1000 bootstrap replicates (Swofford 2003). The MP tree showed that *A. eriantha* was sister to the group including the *A. rufa*, *A. deliciosa*, and *A. chinensis* (Figure 1).

**Figure 1.**
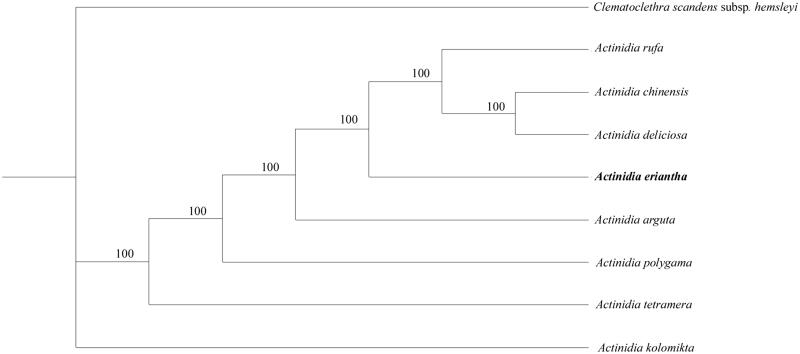
Phylogenetic position of *Actinidia eriantha* in *Actinidia* as inferred by MP analyses of chloroplast genome sequences. Numbers above the lines indicate the maximum likelihood bootstrap value >50% for each clade. Accession Numbers: *Actinidia chinensis* (NC026690), *A. deliciosa* (NC026691), *A. rufa* (NC039973), *A. tetramera* (NC031187), *A. arguta* (NC034913), *A. polygama* (NC031186), *A. kolomikta* (NC034915), and *Clematoclethra scandens* subsp. *hemsleyi* (KX345299).
